# Transurethral injection of autologous muscle precursor cells for treatment of female stress urinary incontinence: a prospective phase I clinical trial

**DOI:** 10.1007/s00192-023-05514-4

**Published:** 2023-04-12

**Authors:** Florian A. Schmid, Jenny A. Prange, Marko Kozomara, Cornelia Betschart, Rosa A. Sousa, Nicolas Steinke, Manuela Hunziker, Fabienne Lehner, Markus Veit, Regina Grossmann, Anna Landsmann, Andreas M. Hötker, Andreas Boss, Deana Mohr-Haralampieva, Daniel Eberli

**Affiliations:** 1 University Hospital Zurich, Department of Urology, University of Zurich, Frauenklinikstrasse 10, 8091 Zurich, Switzerland; 2 University Hospital Zurich, Department of Gynecology, University of Zurich, Zurich, Switzerland; 3 University Hospital Zurich, Clinical Trial Center, University of Zurich, Zurich, Switzerland; 4 University Hospital Zurich, Institute of Diagnostic and Interventional Radiology, University of Zurich, Zurich, Switzerland

**Keywords:** Regenerative therapy, Precursor cells, External urinary sphincter, Minimally invasive, Neuro-muscular electromagnetic stimulation

## Abstract

**Introduction and hypothesis:**

The purpose was to investigate the safety and feasibility of transurethral injections of autologous muscle precursor cells (MPCs) into the external urinary sphincter (EUS) to treat stress urinary incontinence (SUI) in female patients.

**Methods:**

Prospective and randomised phase I clinical trial. Standardised 1-h pad test, International Consultation on Incontinence Questionnaire-Urinary Incontinence Short Form (ICIQ-UI-SF), urodynamic study, and MRI of the pelvis were performed at baseline and 6 months after treatment. MPCs gained through open muscle biopsy were transported to a GMP facility for processing and cell expansion. The final product was injected into the EUS via a transurethral ultrasound-guided route. Primary outcomes were defined as any adverse events (AEs) during follow-up. Secondary outcomes were functional, questionnaire, and radiological results.

**Results:**

Ten female patients with SUI grades I–II were included in the study and 9 received treatment. Out of 8 AEs, 3 (37.5%) were potentially related to treatment and treated conservatively: 1 urinary tract infection healed with antibiotics treatment, 1 dysuria and 1 discomfort at biopsy site. Functional urethral length under stress was 25 mm at baseline compared with 30 mm at 6 months’ follow-up (*p*=0.009). ICIQ-UI-SF scores improved from 7 points at baseline to 4 points at follow-up (*p*=0.035). MRI of the pelvis revealed no evidence of tumour or necrosis, whereas the diameter of the EUS muscle increased from 1.8 mm at baseline to 1.9 mm at follow-up (*p*=0.009).

**Conclusion:**

Transurethral injections of autologous MPCs into the EUS for treatment of SUI in female patients can be regarded as safe and feasible. Only a minimal number of expected and easily treatable AEs were documented.

## Introduction

More than 400 million people worldwide suffer from urinary incontinence (UI), whereas the ratio between women and men is three to one [1]. Stress urinary incontinence (SUI) is the most predominant form of UI [2, 3]. Approximately 50% of the female population over 45 years and around 20% of men after 70 years of age live with SUI [4, 5]. Patients who complain about involuntary loss of urine may experience an impaired quality of life owing to reduced employment rates, earlier institutionalisation, social isolation, recurrent urinary tract infections and other co-morbidities such as psychological disorders [6, 7]. In addition, the ever-increasing health care costs of UI reveal the problem of an unmet medical need [8, 9]. The aetiology of SUI is manifold, whereas an older age and a higher body mass index (BMI) are known to be the most important risk factors [3]. Further, multiple pregnancies with vaginal deliveries, pelvic surgery, or radiation therapy as well as the menopause with the accompanying hormonal changes are associated with a higher probability of SUI [10]. Nevertheless, the physiology of pelvic floor function including UI is complex and not yet entirely understood [11]. Therapy forms are divided into three different categories: conservative, pharmacological and surgical [12, 13]. However, none of the therapies treats the underlying pathophysiological aetiology of SUI. The option of a regenerative approach by using autologous muscle precursor cells (MPCs) to strengthen the external urinary sphincter (EUS) is a promising and minimally invasive therapy option [14–16]. It was our aim to investigate the safety and feasibility of a novel tissue-regenerative approach to treating SUI in female patients with a transurethral injection therapy using autologous MPCs in combination with neuro-muscular electromagnetic stimulation (NMES) in a phase I clinical trial.

## Patients and methods

We conducted a prospective and randomised phase I clinical trial using ultrasound-guided injections of autologous MPCs into the EUS. The aim of the study was to assess the safety and feasibility of this autologous cell therapy for the treatment of SUI in female patients. The local ethics committee (KEK-ZH-Nr. 2014-0547, BASEC Nr. PB_2017-00621) and Swissmedic (Ref. Nr. 2014TpP1009) both approved the phase I study, and the trial was registered on ClinicalTrials.gov (Identifier: NCT03439527). Data entry was supervised by an external, independent data safety monitoring board and trial execution monitored by the internal, independent Clinical Trial Center. The entire study schedule is included as Table 3 in the Appendix.

### Patient recruitment

Initial recruitment was accomplished by specialist referral, online advertisements (study website www.music2020.ch, Google ads and social media), information flyers or printed media. Volunteers were then contacted by telephone to ensure the requirements of the main inclusion and exclusion criteria. Then, a screening visit to the study site was arranged.

### Inclusion and exclusion criteria

The main inclusion criteria were: female gender; age 20–60 years; a clinical diagnosis of SUI grade ≥I according to the Stamey classification for at least 6 months; and post-void residual (PVR) <100 ml.

The main exclusion criteria were: a history of anti-incontinence or prolapse surgery; a previous diagnosis of urinary tract diseases (cystocele, fistula, congenital abnormality or interstitial cystitis); UUI; adult enuresis; urodynamically proven detrusor instability or detrusor–sphincter–dyssynergia (DSD); hyposensitive and/or acontractile detrusor; urethral stenosis; a history of urogenital cancer or pelvic radiotherapy; pregnancy or <12 months postpartum; and unstable systemic, neurological disease or genetically determined muscular disease.

Objective and subjective assessments were performed during the screening (baseline) visit to guarantee that the female patients met the strict inclusion and exclusion criteria. The screening visit included the acquisition of personal medical history, physical examination, vital signs, blood tests, pregnancy test, hypersensitivity test to the injection solution (subcutaneous injection), uroflowmetry, ultrasound of the bladder (including measurement of the PVR) and the kidneys, 1-h pad test, incontinence questionnaire (International Consultation on Incontinence Questionnaire-Urinary Incontinence Short Form [ICIQ-UI-SF]), urodynamic study and magnetic resonance imaging (MRI) of the pelvis.

### One-hour pad test

The testing protocol was accomplished according to the standardised International Continence Society 1-h pad test [17].

### Urodynamic study

The urodynamic study was performed according to the International Continence Society (ICS) standards. To conduct urodynamic investigations, a multichannel urodynamic system (Laborie Medical Technologies Corp., Toronto, Canada) was used. Patients were assessed in a sitting position. UDI comprised same-session repeat filling cystometry, pressure flow study and a resting as well as a stress urethra pressure profile. The bladder was filled with a body-temperature (36°C) 0.9% sodium chloride solution at a speed of 20–30 ml per minute. For simultaneous measurements of vesical and abdominal pressure a 7-French transurethral and rectal latex-free, single-use catheter (T-DOC®, Air-Charged Dual and Abdominal catheter, Laborie Medical Technologies Corp., Toronto, Canada) was used. For the UPP measurement the bladder was prefilled with 150 ml and the catheter was withdrawn by a mechanical device at a rate of 1 mm/s. The urethral closure pressure (UCP) profile, the maximum urethral pressure (MUP), the maximum urethral closure pressure (MUCP) and the functional urethra length (FUL) were obtained from the UPP.

### Magnetic resonance imaging of the pelvis

Magnetic resonance imaging of the pelvis was performed at a field strength of 3 Tesla (Siemens MAGNETOM Skyra, Siemens Healthineers, Erlangen, Germany) using standard high-resolution T2-weighted turbo spin echo sequences with and without fat suppression and T1-weighted turbo spin echo sequences. An 18 Ch body coil was used for signal reception. The longest diameter of the sphincter muscle was measured on pre- and post-therapeutic examinations and all examinations were screened for possible areas of necrosis or tumour.

### Muscle biopsy

To obtain an appropriate muscle specimen for MPC isolation and expansion, an open surgical biopsy coming from a striated skeletal muscle was performed in analgosedation on either the left or the right lower leg in each of the patients. After weighing the muscle biopsy in a cooled Falcon tube containing biopsy medium, it was sent to the good manufacturing practice (GMP) facility.

### Cell isolation, expansion and final product

Manufacturing of the MPCs was performed according to GMP standards in the clean room facility of the Wyss Translational Center Zurich (manufacturer authorisation number 512701-102673231). The manufacturing process consisted of MPC isolation, MPC expansion and final product preparation, including strict in-process and product release controls. With confirmed compliance, 80–100 × 10^6^ MPCs were mixed with the injection solution, loaded into a syringe and transported under controlled temperature conditions to the hospital.

### Injection of MPCs

The injection was performed according to a standardised procedure under general anaesthesia and in full lithotomy position. Before starting the operation, the batch number from the GMP facility was double-checked with the patient’s study ID. After insertion of a vaginal ultrasound probe (BK Medical®, Endocavity 3D 8838) the anatomy was assessed, and the EUS identified. Then, a transurethral catheter (Rüsch®, SupraCath Silicone Ch10) was inserted. The implantation of the cooled final product (autologous MPCs) was performed through transurethral injections under ultrasound guidance into the horseshoe-configured anterior and anterolateral muscle tissue of the EUS, surrounding the proximal part of the urethra. At the end of the procedure, all devices were removed except for the indwelling catheter.

### Follow-up visits

The first follow-up visit was on the first postoperative day. After removal of the catheter in the morning, patients were seen for a physical examination, uroflowmetry and ultrasound including PVR measurement. Further visits in the ambulatory setting were arranged 1, 3 and 6 months after the intervention. The follow-up visit at 6 months was composed of an additional urodynamic study and MRI of the pelvis as a comparison with the baseline visit.

### Neuromuscular electromagnetic stimulation

After the injection, the study nurse unveiled the 1:1 randomisation to the two treatment groups of either MPC vs MPC + NMES. NMES included two sessions per week for 20 min for 6 weeks with a total of 12 sessions on a BioCon-2000W™ chair (Marly Products®, Germany). Patients used its predefined program for SUI and the percentage of intensity was individually ramped up to the subjective pain threshold as instructed by the study doctors.

### Primary and secondary outcomes

To analyse the safety and feasibility of our therapy, primary outcomes were defined as any adverse event (AE) during the follow-up period. AEs were classified according to Clavien–Dindo grade I–V. Secondary outcomes were chosen as objective and subjective outcome parameters. Objective parameters were uroflowmetry, PVR and the 1-h pad test. Further, the results from the urodynamic study included maximal bladder capacity, bladder compliance, presence and amount of leakage, maximal urethral closure pressure (MUCP) and functional urethra length (FUL) at rest and under stress (continuous coughing). Subjective parameters were patient-reported outcomes (PROMs) as in the evaluation of the filled-in questionnaires (ICIQ-UI-SF [0–21 points] including amount of pad usage per day, Visual Analogue Scale [VAS 0–10 points] for degree of suffering and quality of life) and necessity of subsequent incontinence surgery during follow-up. Anatomical aspects (aberrant tissue or necrosis formation) and the diameter of the EUS were measured before treatment and at EOS on the MRI of the pelvis in every patient.

### Statistical analysis

The statistical analysis was outsourced to an independent company (Hemex AG, Liestal, Switzerland) and performed using *R*, version 4.1.1 [18]. Baseline characteristics are presented by means or medians including ranges and standard deviations (SD). Primary outcomes as in AEs are reported in a descriptive manner. Paired Wilcoxon signed-rank test was used to compare medians of secondary outcomes between baseline and follow-up visits. Owing to the limited number of patients included in this cohort, the trial was not powered for the analysis of secondary outcomes and therefore these results need to be interpreted with caution. Further, no subgroup analysis was performed to investigate the results of the randomised groups (MPC vs MPC + NMES). Statistical significance was regarded as *p*<0.05.

## Results

Ten female patients were included in the study, 9 of whom received treatment and completed all follow-up visits during the study period between January 2020 and September 2021. One patient had to be excluded in between muscle biopsy and injection owing to an erroneous interpretation of a supposedly contaminated sterility test of the cell product by an external laboratory. The test was later repeated, and the sterility of the cell product was confirmed retrospectively.

### Baseline characteristics

**Fig. 1:**
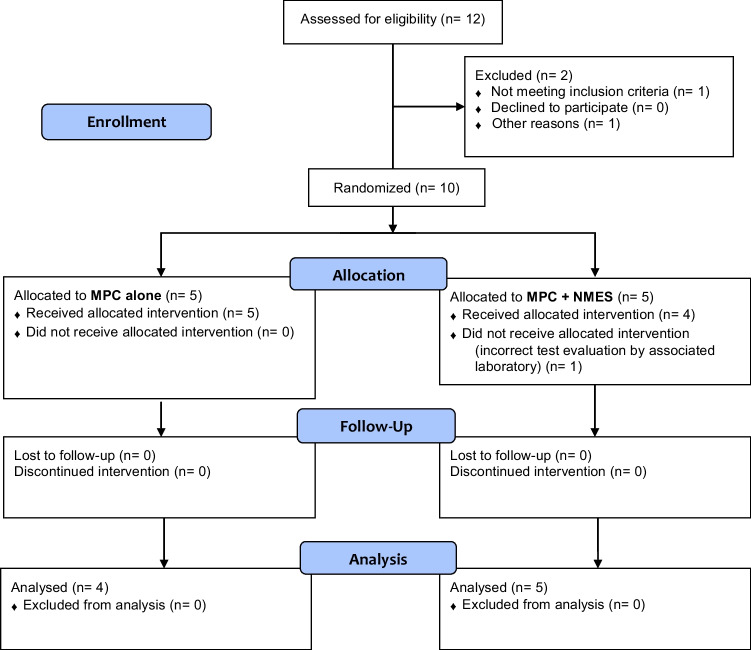
CONSORT flow diagram of patients screened, included, randomised, treated and followed up during a phase I clinical trial with an intention-to-treat approach. *MPCs* muscle precursor cells, *NMES* neuromuscular electromagnetic stimulations

Patients had mild to intermediate SUI (*n* = 8 with SUI grade I, *n* = 1 with SUI grade II), a median age of 45 years (range: 32–58 years) and a median BMI of 24 kg/m^2^ (21.0 – 29.4 kg/m^2^). After MPC injection, patients were subsequently randomised into groups of either MPC alone (*n*=5) or MPC + NMES (*n*=4). Please find the study flow-chart and patient characteristics presented as Fig. 1 and Table 1 respectively.

**Table 1:** Patient characteristics stratified by treatment group (MPC vs MPC + NMES). Data presented as mean (SD) or *n* (%)

	MPC	MPC + NMES
*n*	5	4
Age (years)	43.60 (10.69)	48.25 (6.85)
Height (cm)	164.40 (8.88)	167.50 (3.32)
Weight (kg)	61.60 (3.44)	74.50 (10.34)
BMI (kg/m^2^)	22.86 (1.77)	26.52 (3.28)
Smoking		
No	4 (80.0)	3 (75.0)
Yes	0 (0.0)	0 (0.0)
Former	1 (20.0)	1 (25.0)
SUI grade		
I	5 (100.0)	3 (75.0)
II	0 (0.0)	1 (25.0)
III	0 (0.0)	0 (0.0)
Previous therapy (non-surgical)	3 (60.0)	1 (25.0)

*MPC* muscle precursor cell, *NMES* neuromuscular electromagnetic stimulations, *SD* standard deviation (±), *BMI* body mass index, *SUI* stress urinary incontinence

### Adverse events

There has been no reaction to the injection solution after subcutaneous test injection. Neither an unexpected reaction or AE after biopsy retrieval at the lower limb nor after transurethral injection of the final product into the EUS was seen. Further, no relevant complications or severe AEs (SAEs) were documented during follow-up. However, 8 AEs were registered, of which 3 (37.5%) were expected and potentially related to the treatment procedure. One urinary tract infection (UTI) was diagnosed 3 weeks after injection of MPCs and was successfully treated with a single dose of oral antibiotic treatment. Burning micturition (dysuria) due to decatheterisation without presence of an UTI was treated conservatively in 1 further patient. The other UTIs happened later during the follow-up period (>4 weeks after injection). Further, 1 patient expressed slight discomfort at the biopsy site during intense sport activities and was treated conservatively. Finally, one ankle joint distortion was documented independent of the treatment. To summarise, all complications were ≤ grade II according to Clavien–Dindo classification (Table 2).

**Table 2:** Presentation of adverse events (*AEs*) during treatment and follow-up according to treatment groups

Category of AEs	AEs	Number of AEs before/after injection of MPCs		Total number of AEs/related to treatment	Percentage of all AEs/related to treatment
		MPC group	MPC + NMES group		
LUTS	UTI	0/0	1/3	4/1	50/12.5
	Dysuria	0/1	0/0	1/1	12.5/12.5
Other	Discomfort at biopsy site	0/0	0/1	1/1	12.5/12.5
	Ankle joint distortion	1/1	0/0	2/0	25/0
Total		1/2	1/4	8/3	100/37.5

*LUTS* lower urinary tract symptoms, *UTI* urinary tract infection, *MPCs* muscle precursor cells, *NMES* neuromuscular electromagnetic stimulation

### Functional outcome parameters

**Fig. 2:**
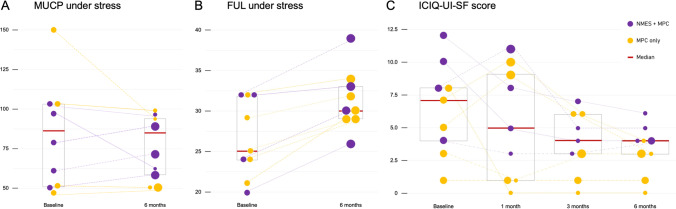
Baseline and follow-up visits compared. **A** Maximal urethral closure pressure (*MUCP*) under stress (−8 cmH_2_0, 95% CI: −9 to 28.5, *p*=0.859). The unit for the y-axis is cmH_2_O. **B** Functional urethral length (FUL) under stress (+5 mm, 95% CI: 2.5 to 7, *p*=0.009). The unit for the y-axis is mm. **C** Change in International Consultation on Incontinence Questionnaire (*ICIQ-UI-SF*) score (−3 points, 95% CI: −7 to −2.5, *p*=0.035). The unit for the y-axis is ICIQ-UI-SF score. *MPCs* muscle precursor cells, *NMES* neuromuscular electromagnetic stimulations

No relevant PVR (<50ml) was documented with sonography at baseline and follow-up visits in all patients. Median MUCP at rest decreased from 91 cmH_2_O at baseline to 75 cmH_2_O at 6 months’ follow-up (difference: −16 cmH_2_0, 95% CI: −17 to 39, *p*=0.624). Median FUL at rest was 35 mm at baseline compared with 34 mm at 6 months’ follow-up (difference: −1 mm, 95% CI: −5 to 4, *p*=0.343). Median MUCP under stress was 79 cmH_2_O at baseline compared with 71 cmH_2_O at 6 months’ follow-up (difference: −8 cmH_2_0, 95% CI: −9 to 28.5, *p*=0.859; Fig. 2A). Median FUL under stress was statistical significantly shorter, with 25 mm at baseline compared with 30 mm at 6 months’ follow-up (difference: +5 mm, 95% CI: 2.5 to 7, *p*=0.009; Fig. 2B). Median maximum bladder capacity increased from 610 ml at baseline to 670 ml at follow-up (difference: +60 mL, 95% CI: −45 to 140, *p* = 0.343). Average micturition volume between baseline and follow-up visit was similar with 558 mL and 575 mL, respectively (difference: +17 mL, 95% CI: −351 to 148, *p* = 0.106).

### Pad use

At baseline, 2 patients reported using no pads during daily routine, whereas 6 patients reported using 1 pad/day, and 1 patient using 2 pads/day. At the 6-month follow-up, 1 out of the 6 patients who needed 1 pad/day reported no longer needing any pads, 5 patients still used 1 pad/day, and the patient who needed 2 pads/day had reduced to 1 pad/day 6 months after MPC treatment. Three patients had a positive 1-h pad test and lost urine at baseline visit (range: +1 to +6 g, median: +3 g), whereas pads stayed dry in all other patients. At follow-up, the 1-h pad test was positive in 1 patient (+3 g at baseline and +1 g at follow-up), whereas no increase in pad weight was found in the rest of the cohort.

### ICIQ-UI-SF questionnaires

The ICIQ-UI-SF questionnaires showed a statistically significant improvement in scores from a median of 7 points at baseline to a median of 4 points at 6 months’ follow-up (difference: −3 points, 95% CI: −7 to −2.5, *p*=0.035; Fig. 2C).

### MRI of the pelvis

**Fig. 3:**
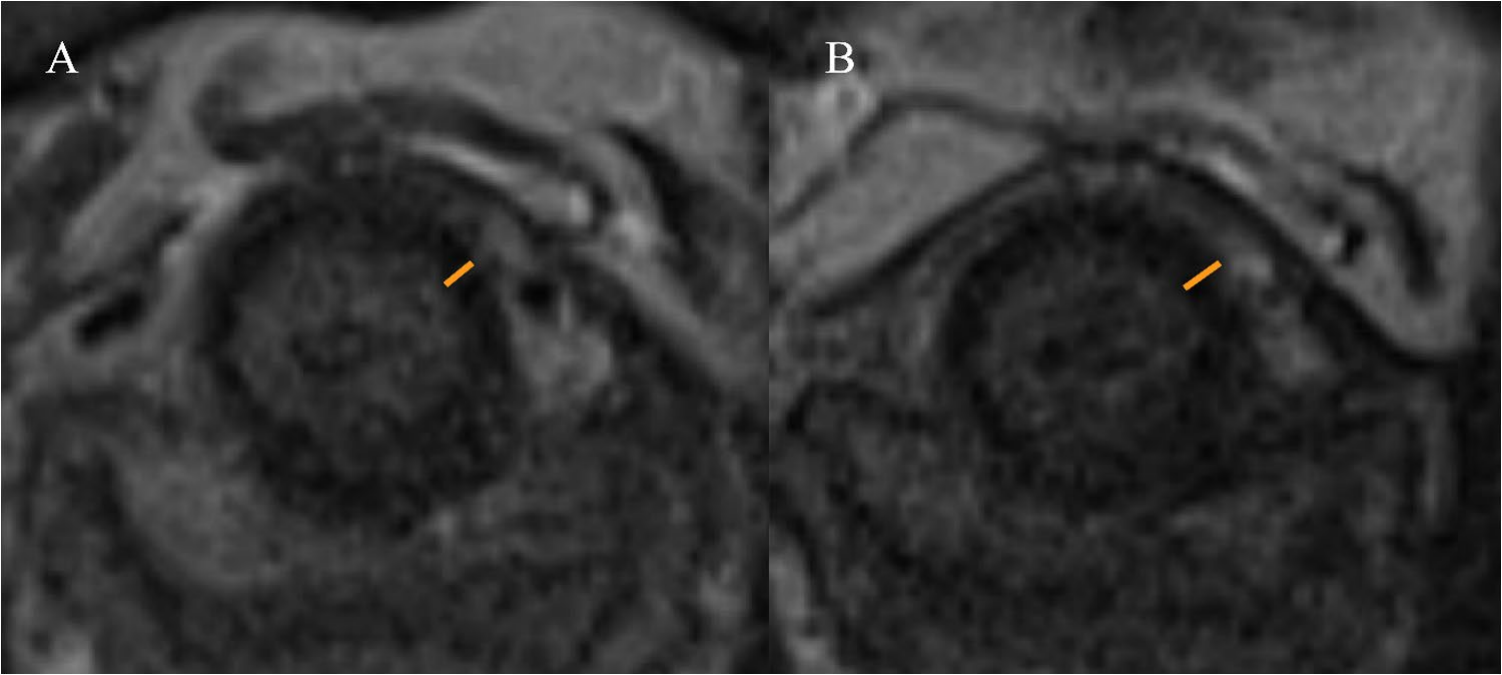
MRI of the pelvis with a T2-weighted sequence in axial and sagittal orientation for the anatomical analysis of the external urinary sphincter (*EUS*) diameter, no presence of necrosis or tumour at **A** baseline and **B** follow-up

The evaluation of MRI of the pelvis revealed no evidence of aberrant tissue formation (i.e. tumour) or necrosis. The diameter of the EUS was measured with a statistically significantly larger median of 1.8 mm at baseline and 1.9 mm at follow-up (difference: +0.1 mm, 95% CI: 0.10 to 0.25, *p*=0.009; Fig. 3).

## Discussion

Our phase I clinical trial demonstrates that the implantation of autologous MPCs in combination with or without NMES for the treatment of SUI grade ≥I in women is safe and feasible. Therapy was well tolerated by patients and no relevant or unexpected AEs and no need for consecutive surgical or interventional treatments were documented (all complications Clavien–Dindo grade ≤II). Further, our analysis demonstrated an objective improvement of the median FUL under stress in the urodynamic study 6 months after injection of autologous MPCs into the EUS. In addition, PROMs evaluated by questionnaires improved with a lower median ICIQ-UI-SF score at 6 months after the intervention, suggesting a better quality of life in these patients. All patients with a positive 1-h pad test at baseline improved at follow-up with 2 out of 3 patients being completely dry. Finally, the analysis of the MRI of the pelvis revealed an increase in EUS diameter. Most importantly, no evidence of cell transformation (i.e. tumour tissue or necrosis) at the end of follow-up was found radiologically. The decrease in MUCP and FUL values at rest comparing from before with after treatment was not statistically significant. However, all results need to be further elucidated in larger patient cohorts during prospective phase II and III trials. To investigate the overall regenerative potential of this approach, patients are further included in a long-term follow-up study for the analysis of safety and efficacy up to 5 years after treatment.

For more than two decades, there has been an immense research effort to ameliorate the treatment of SUI with a regenerative approach [19]. Mesenchymal (bone marrow), adipose (ADSCs) and muscle-derived stem cells (MDSCs) have been further pursued, whereas the usage of embryonic stem cells brings along ethical concerns and a higher risk of tumour formation [20]. Researchers were increasingly able to successfully translate in vitro results into animal models that demonstrated promising functional outcomes [21, 22]. Later clinical trials have shown similar short-term and better long-term functional results compared with other minimally invasive procedures (such as bulking agents), mostly using adult stem cells originating from muscle or fatty tissue [23]. During clinical trials even autologous pure myoblasts isolated from biceps muscle samples or minced skeletal muscle tissue were used in combination with or without electrical stimulation of the lower pelvis for the treatment of SUI in the past [14, 24, 25]. Until today, the development of cell therapies has mostly been limited to preclinical and human feasibility studies, in which it is difficult to monitor cell fate and objectively rate functional outcomes other than subjective PROMs [26]. In comparison with most other studies, we have completed follow-up visits with a urodynamic study and MRI of the pelvis to maximise the objective evaluation of functional and anatomical outcomes in comparison with baseline parameters.

Results from studies investigating other therapy approaches with transurethral injections of autologous MDSCs have shown them to be safe and feasible without any relevant AEs or SAEs. Dose-ranging studies using MDSCs (1–200 million cells) by Chancellor and his group [19, 20] demonstrated that higher dose groups tended to have a more favourable effect on continence regarding pad tests, stress leaks and questionnaire results without any complications during treatment and follow-up [15, 27]. Jankowski and colleagues performed the only double-blind, randomised and placebo-controlled multicentre clinical trial evaluating the safety and efficacy of MDSCs for treating SUI in women [16]. Although treatment was well tolerated, with no SAEs or discontinuation of treatment due to AEs, the trial had to be stopped after enrolment of 61% of patients owing to an unexpectedly high placebo response rate (90%). The conclusion suggests that composite outcomes were responsible for high placebo rates and may have concealed a potential treatment effect. Results from a French investigation in 12 female patients with SUI has shown that more than 80% were either dry or improved on pad tests and that quality of life was better in half of the patients 1 year after autologous MDSC injection isolated from the deltoid muscle [28]. No relevant AEs were reported other than three occasional UTIs, which were treated with antibiotics and resolved completely. The longest follow-ups were presented by Iranian (3 years) and Polish (2 years) studies [29, 30]. Sharifiaghdas et al. investigated 10 female patients with SUI due to trauma of the lower pelvis who were treated with transurethral autologous MDSC injections [29]. They reported that 7 were either cured or improved on 1-h pad tests or MUCP measurements in urodynamic studies at the end of follow-up. Stangel-Wojcikiewicz and colleagues treated 16 female patients with autologous MDSCs suffering from SUI and presented a complete or partial improvement in 12 patients (75%) and a significantly improved quality of life with no SAEs or complications until the end of follow-up [30]. These published results are comparable with our study outcomes regarding safety and feasibility. Likewise, we did not encounter any relevant AEs that could potentially hamper the regenerative therapy approach of autologous MPC injections for the treatment of SUI in women. Additionally, our cell product showed promising secondary outcomes: functional, quality of life and anatomical measurements resulted in ameliorated FUL under stress (+5 mm), improved ICIQ-UI-SF scores (−3 points) and an increased EUS diameter (+0.1 mm) at the end of follow-up at 6 months postoperatively respectively.

It is worth mentioning that different muscle-derived cell populations have shown a potentially different impact on functional regeneration and their modes of action—some through paracrine effects, others through direct integration into the damaged tissue. Importantly, the main therapeutic effect in our patient population is achieved by MPC-induced muscle regeneration of the EUS. The additional usage of NMES may be a potential advantage for an ameliorated functional outcome owing to additional growth stimulation through training of the pelvic musculature. However, a subgroup analysis to investigate the therapeutic effect of MPC + NMES vs MPC alone was not feasible in the underlying study owing to limited patient numbers. Altogether, all these results underline the potential of a regenerative therapy approach to the treatment of SUI in women.

Our functional results need to be interpreted with caution as this was a phase I trial investigating solely safety and feasibility. Owing to the limited number of patients and the associated lack of statistical power, no subgroup analysis regarding the randomisation into MPC vs MPC + NMES was performed. Further, only 3 out of 9 patients had a positive 1-h pad test at baseline. Therefore, the efficacy and durability of the treatment need to be confirmed with larger patient cohorts and longer follow-up periods in specifically designed, accordingly powered and prospectively performed phase II and III trials.

## Conclusion

The transurethral ultrasound-guided injection of autologous MPCs into the EUS for the treatment of SUI in female patients can be regarded as safe and feasible. Only a minimal number of expected AEs were documented, and all AEs were easily treatable and healed without sequelae. No severe or unexpected AEs were diagnosed. At the same time, promising overall functional and anatomical outcomes, as well as quality of life measurements, were found.

## Appendix

**Table 3:** Study schedule

Study periods	Screening (baseline)	Biopsy	Treatment phase		Post-treatment phase			EOS
Visit	1	2	3	4	5	6	7	
Day	7–14 days prior to biopsy	−21±7 days	0	2 days ± 1 day	1 month ± 1 week	3 months ± 1 week	6 months ± 1 week	Study termination + 1 week
Subject information and informed consent	x							
Randomisation				x				
Demographics	x							
Medical history	x							
Inclusion/exclusion criteria	x							
Physical examination	x	x^e^	x^f^	x				x^g^
Vital signs	x	x	x	x	x	x	x	x^g^
Blood sampling tests^a^	x	x^e^	x^f^		x	x	x	x^g^
Hypersensitivity test	x							
Pregnancy tesb	x		x					
Lower leg biopsy		x						
Resting ECG		x^e^	x^f^					
Chest X-ray		x^e^	x^f^					
Implantation of MPCs			x					
Electromagnetic stimulation^b^				x	x	x		
Ultrasound kidney, bladder	x			x	x	x	x	
Post-void residual volume	x			x	x	x	x	
Uroflowmetry	x			x	x	x	x	
Urodynamic evaluation	x					x	x	
1-h pad test	x				x	x	x	
Incontinence score (questionnaire)	x				x	x	x	
Quality of life score	x				x	x	x	
Degree of suffering (VAS)	x				x	x	x	
Rate of subsequent incontinence surgery							x	
Concomitant medication and treatment^c^	x	x	x	x	x	x	x	
Adverse events		x	x	x	x	x	x	x^g^
MRI pelvis^c, d^	x^d^				x^d^	x^d^		

*ECG* electrocardiography, *MPCs* muscle precursor cells, *VAS* Visual Analogue Scale, *MRI* magnetic resonance imaging, *NMES* neuro-muscular electromagnetic stimulation

^a^Blood chemistry, haematology

^b^Magnetic stimulation (“MPC + NMES group” only), from day 2 to week 6, twice a week, 50% of the cohort by randomisation

^c^Standard medications for general and spinal anaesthesia respectively will be collected in the patient history file, but they will not be entered into the case report form

^d^Additional substudy (optional)

^e^Biopsy will be performed under general anaesthesia. Physical examination, blood sampling tests, ECG and X-ray will be performed according to the guidelines of the University Hospital Zurich for pre-surgical evaluation for general anaesthesia

^f^Implantation of MPCs will be performed under general or spinal anaesthesia. Physical examination, blood sampling tests, ECG and X-ray will be performed according to the guidelines of the University Hospital Zurich for pre-surgical evaluation for general and spinal anaesthesia respectively

^g^Visit 7 may be combined with EOS. If not combined (i.e. for patients who dropped out or withdrew consent earlier), please note that for EOS, the following is mandatory: performance of physical examination, vital signs and (safety), blood sampling tests as well as assessment of concomitant medication and treatment and adverse events
